# The Impact of Antimalarial Use on the Emergence and Transmission of *Plasmodium falciparum* Resistance: A Scoping Review of Mathematical Models

**DOI:** 10.3390/tropicalmed2040054

**Published:** 2017-10-15

**Authors:** Aleisha R. Brock, Carole A. Gibbs, Joshua V. Ross, Adrian Esterman

**Affiliations:** 1 School of Nursing and Midwifery, Division of Health Sciences, University of South Australia, Adelaide, SA 5000, Australia; 2 Library, University of South Australia, Adelaide, SA 5000, Australia; carole.gibbs@unisa.edu.au; 3 School of Mathematical Sciences, The University of Adelaide, Adelaide, SA 5005, Australia; joshua.ross@adelaide.edu.au; 4 Sansom Institute for Research Health, University of South Australia, Adelaide, SA 5000, Australia; adrian.esterman@unisa.edu.au; 5 Australian Institute of Tropical Health and Medicine, James Cook University, Smithfield, QLD 4878, Australia

**Keywords:** scoping review, malaria, *Plasmodium falciparum*, mathematical model, antimalarial resistance, antimalarial treatment, subtherapeutic, drug quality

## Abstract

The emergence and transmission of resistance to antimalarial treatments continue to hamper malaria elimination efforts. A scoping review was undertaken regarding the impact of antimalarial treatment in the human population on the emergence and transmission of *Plasmodium falciparum* resistance, to (i) describe the use of mathematical models used to explore this relationship; (ii) discuss model findings; and (iii) identify factors influencing the emergence and transmission of resistance. Search strategies were developed and deployed in six major databases. Thirty-seven articles met the eligibility criteria and were included in the review: nine articles modeled the emergence of resistance, 19 modeled the transmission of resistance, and nine modeled both the emergence and transmission. The proportion of antimalarial use within the population and the presence of residual drug concentrations were identified to be the main predictors of the emergence and transmission of resistance. Influencing factors pertaining to the human, parasite and mosquito populations are discussed. To ensure the prolonged therapeutic usefulness of antimalarial treatments, the effect of antimalarial drug use on the emergence and transmission of resistance must be understood, and mathematical models are a useful tool for exploring these dynamics.

## 1. Introduction

Malaria is considered to be one of the world’s most significant infectious diseases in terms of morbidity and mortality, and is endemic in 91 countries. In 2015, there were approximately 212 million cases of symptomatic malaria worldwide, resulting in 429,000 deaths. Prevalent in parts of Africa, *Plasmodium falciparum* malaria accounts for 90% of the global cases and 92% of global deaths, the majority of which occur in children under five years of age [1]. It is caused by an infection of the red blood cells with plasmodia parasites, with the disease lifecycle occurring in two phases: one within the human host, and one within the female *Anopheles* species mosquito vector.

The emergence and transmission of resistance to antimalarial treatment continues to hamper malaria elimination efforts. Widespread resistance to antimalarials occurs in two phases: first, resistance must emerge within the plasmodia population in order to confer a survival advantage in the presence of drugs; and secondly, resistance must be transmitted through both the human and mosquito populations, which then becomes widespread [2], resulting in treatment failure or death.

The emergence of antimalarial resistance is caused by a random genetic event within plasmodia, whereby a point mutation, or a set of mutations, occurs that may or may not give rise to resistance [3], irrespective of antimalarial treatment [4]. Mutations affording resistance to antimalarial drug(s) can require one or more mutations, which can be linked or unlinked [5]. Intermediate or increasing levels of resistance have been observed through the acquisition of multiple mutations, as with sulfadoxine-pyrimethamine [6] and combination therapy resistance [5].

The survival of resistant plasmodia parasites is determined by natural selection and drug selection. Natural selection refers to the elimination of mutant (including resistant) parasites from direct competition with wild parasites, if the mutation required to produce resistance results in a reduction in fitness compared to competing strains [7]. Drug selection refers to the ability of the parasites to survive in the presence of antimalarial drug, where drug-sensitive parasites will be eliminated, and parasites with resistant-conferring mutations will dominate the infection (competitive release) [6].

Once a resistant strain has emerged and been selected through natural and drug selection, the transmission of resistant plasmodia is very hard to prevent and can have catastrophic effects on the community. In the initial stages of resistance, treatment with the ineffective antimalarial drug continues within the community, as resistance has yet to be detected. This continued ineffective treatment may result in prolonged parasite clearance or treatment failure. Gametocytes with the advantageous mutation(s) will be transmitted to a mosquito during a blood meal [6]. For transmission of the resistant sporozoite plasmodia from the female *Anopheles* mosquito vector to another human host, the mutant parasite must outcompete other parasite strains in the bottleneck that occurs in the mosquito midgut [8] and survive chromosomal crossover and recombination with the resistant-conferring mutations remaining intact. Chromosomal crossover and recombination can either aid or prevent the continued survival of resistant mutants and occur in the *Anopheles* mosquito intestinal wall [9]. Chromosomal crossing occurs between homologous chromosomes, in which slices of chromosome are exchanged. Genetic recombination is the process by which genetic material is shared between unlinked and linked nuclear genes. The ability to exchange genetic material aids in the survival of plasmodia by increasing population variability, along with the sharing of any resistant gene(s) required for survival in the presence of antimalarials [10]. However, during this time, the mutated genes conferring resistance may be spliced, and resistance lost. The advantage of antimalarial resistance to a parasite is only apparent under drug selection pressure, where drug sensitive parasites are cleared allowing resistant parasites to dominate the infection [6]. Eventually the resistant parasite population will become established within the infected human and mosquito populations.

Mathematical models have been used to explore the dynamics of malaria for over 100 years [11], and have been used to propose strategies well in advance of comprehensive data becoming available [12].

### Study Objective

The objectives of this scoping review are to: (i) to gather and appraise all available literature that feature mathematical models of the emergence and/or transmission of resistance in *P. falciparum* that take into account antimalarial use within the human population; (ii) compare and contrast the predicted impact of antimalarial use on the emergence and transmission of resistance, as identified by the models; and (iii) identify key factors that influence this relationship.

## 2. Review Methodology

A scoping review was carried out using a systematic process defined by Arksey and O’Malley (2005) [13], and further enhanced by Levac and colleagues (2010) [14] and Colquhoun and colleagues (2014) [15]. The six-step framework proposed by Arksey and O’Malley has been followed, which ensures rigor and transparency, with the sixth step omitted for the purposes of this review [13].

### 2.1. Step One: Identifying the Research Question

In the first stage of the review, three research questions were developed:

1. How have mathematical modeling methods been used to assess the impact of antimalarial treatment in the human population on the emergence and transmission of *P. falciparum* resistance?
2. What risk factors have been identified by mathematical models, of the use of antimalarial treatment in the human population on the emergence and transmission of *P. falciparum* resistance?
3. What other factors have been identified to influence this relationship, through the use of mathematical modeling?

### 2.2. Step Two: Identifying Relevant Studies

A comprehensive search of the literature was carried out for work published until the end of August 2016, under the guidance of author CAG. The following electronic databases were searched (in order): Medline, Embase, CINAHL, Web of Science, Cochrane Library and Scopus; and grey literature searches (Google Scholar and Open Grey). The MeSH headings and key search terms, along with the combinations used, are provided in Supplementary Materials Tables S1–S6. During full-text screening, the reference lists of relevant articles were also reviewed. All records were exported and managed within Covidence [16].

### 2.3. Step Three: Study Selection

The articles all proceeded through (i) title and abstract screening; and (ii) full-text screening. The inclusion and exclusion criteria are defined below.

#### 2.3.1. Inclusion Criteria

- Article must be written in English.
- Publishing date until the end of August 2016.
- The analysis section must contain a mathematical modeling-based approach.
- Results must be provided.
- The human malaria species *P. falciparum* must be modeled.
- The human host must be studied in the model, with outputs relevant to the human population provided. Other populations, such as the *P. falciparum* parasite or female *Anopheles* mosquito vector may also be included.
- The model must explore the effect of an antimalarial agent on the emergence and/or transmission (spread) of antimalarial resistance in *P. falciparum*.
- Full text must be available.

#### 2.3.2. Exclusion Criteria

- Mathematical model not defined in the article.
- Human malaria species: *P. vivax*, *P. malariae*, *P. knowlesi*.
- Results section does not discuss the dynamics in regards to the human population.
- No full text available (e.g., conference abstract, embargoed).

### 2.4. Stage Four: Charting the Data

A data collection form was developed to ensure consistency in data collection from the articles. The data were charted (entered into tables) by summarizing each article reviewed by: author, study aim/objective, model design, key findings, recommendations, and our comments/critique. The comments/critique focused on (i) the transparency and reproducibility of the model, given the information provided in the article—for example, are the equations and parameter lists provided? and (ii) the model analysis procedures utilized—for example, whether the findings were validated or whether the model behavior was explored through a sensitivity analysis.

### 2.5. Stage Five: Collating, Summarising, and Reporting the Results

In the final stage of the review, the articles were then categorized as modeling the (i) emergence of antimalarial resistance; (ii) transmission of antimalarial resistance; or (iii) emergence and transmission of antimalarial resistance. The impact of antimalarial use and each category was then assessed and discussed (drug selection pressure), as well as other influencing factors that were taken into account in the model (*P. falciparum*, host immunity, transmission intensity, and vector control).

## 3. Results

### 3.1. Study Selection

**Figure 1:**
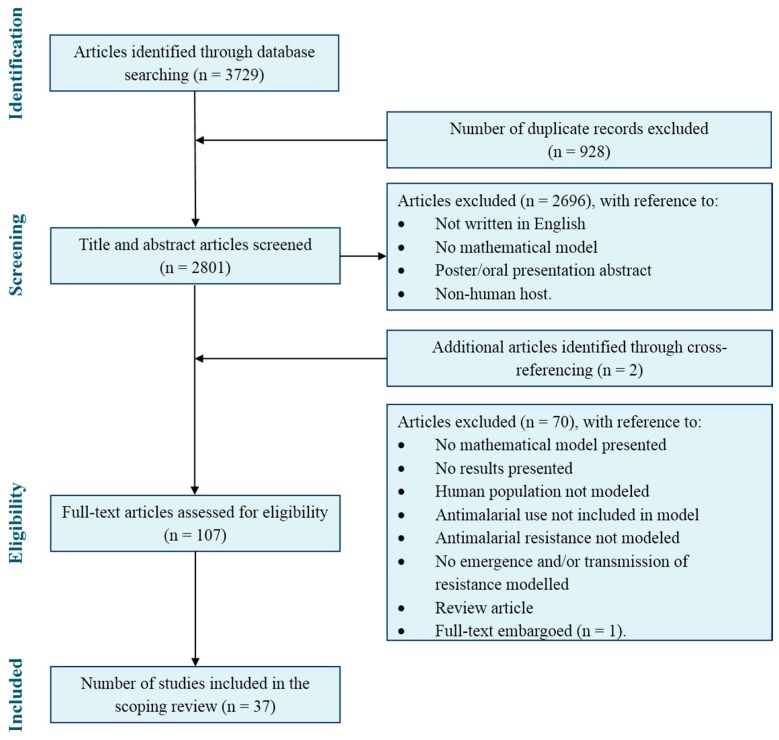
PRISMA flow diagram of the scoping review search and article selection.

Our search identified 2803 potential articles for the scoping review after duplications were removed, of which 37 articles met the inclusion criteria. Validation of article selection was carried out during the title and abstract screening by author AE. Both authors (ARB and AE) had an agreement rate of 97.8%. Of the articles for which there was a conflict, a Yes/No conflict were found in 66.7% of the cases and Maybe/No conflict in the remaining 33.3%. The authors revisited these articles and assigned them accordingly. Full text screening and analysis of results were carried out by author ARB and validated by author JVR. An overview of the study selection process is provided in Figure 1.

### 3.2. Mathematical Modelling Methods

#### 3.2.1. Model Descriptions

Of the 37 articles reviewed, nine modeled the emergence of resistance [8,17,18,19,20,21,22,23,24], 19 modeled the transmission of resistance [6,25,26,27,28,29,30,31,32,33,34,35,36,37,38,39,40,41,42] and nine modeled both [43,44,45,46,47,48,49,50,51]. A summary of model elements and features is provided in Table 1.

The most common form of mathematical modeling was deterministic modeling (25/37, 68%), with stochastic modeling used in nine articles (24%), and a combination of deterministic and stochastic modeling techniques used in three (8%) articles. Deterministic models contain no random probabilistic elements, providing constant model outcomes for a specific set of initial values and parameters; unlike stochastic models, which sample from a probability distribution for each parameter and allow for random variation.

Twelve (32%) articles modeled the dynamics within the human and mosquito populations, predominantly to take into account transmission. The human population alone was modeled in 11 (30%) articles; interestingly, none of these models explored the dynamics of the emergence of resistance. The human and *P. falciparum* populations were modeled in nine (22%) articles; and the remaining five (14%) articles modeled the human, mosquito and *P. falciparum* populations.

The majority of the articles used the literature to inform parameter values (28/37, 76%), whereas nine articles used field, clinical or laboratory observations from Tanzania, Uganda, Sudan, Burkino Faso, Cambodia, and Thailand.

The model equations were provided in 86% (32/37) of the articles (whether in text or supplementary), and a list of parameter values (tabulated or in text) were provided in 86% (32/37) of the articles. The model assumptions were outlined in every article reviewed (100%). An analysis of model behavior in the form of assessing the sensitivity of the model outputs to parameter values (sensitivity analysis) and/or validating the model outputs through a comparison of key outputs to literature, laboratory or clinical data observations (model validation), were presented in 62% (23/37) of articles.

#### 3.2.2. Antimalarial Resistance

Antimalarial resistance to 11 monotherapy treatments were modeled (emergence: three treatments; transmission: seven treatments; emergence and transmission: five treatments), with five articles not specifying the type of monotherapy treatment (emergence: two articles [18,22]; transmission: three articles [25,27,34]). Resistance to combination therapies were modeled in 13 articles: artemisinin-based combination therapy (ACT) resistance in two articles, sulfadoxine-pyrimethamine (SP) resistance in seven articles, and four articles failed to specify the combination therapy to which resistance was present within the population [18,22,25,34]. There were 15 articles that did not specify the antimalarial treatment to which resistance existed. Partner-drug resistance was explored in five articles. Differing levels of resistance (partial, full and/or varying numbers of mutations affording resistance) were modeled in 11 articles.

#### 3.2.3. Antimalarial Treatment

Drug selection pressure was modeled through the use of antimalarial treatments, treatment strategies, and antimalarial properties. The type of antimalarial treatments modeled in the articles included nine different antimalarial monotherapy treatments and 16 different antimalarial combination therapies. Antimalarial treatment was not specified in 16 articles, with an additional five articles not specifying the type of monotherapy treatment, and four articles not specifying the type of combination therapy modeled. Multiple treatments (monotherapy and/or combination therapy) were modeled in 16 articles: 11 articles modeled monotherapies and combination therapies [6,8,18,22,24,25,34,36,41,43,49]; two articles modeled more than one monotherapy treatment [42,43]; and six articles modeled more than one combination therapy [8,24,35,36,39,42]. Antimalarial treatment strategies were explored in transmission and emergence and transmission models, namely the use of intermittent preventive treatment (IPT) in four articles [6,39,41,49], mass drug administration (MDA) in three articles [35,36,49], and mass screening and treatment (MSAT) in one article [36]. Antimalarial properties, such as the drug half-life, residual drug levels, dosage and treatment compliance, were modeled in 12 articles.

#### 3.2.4. Potential Influencing Factors

Other factors included in the mathematical models that have the potential to influence the relationship between resistance and antimalarial use, were *P. falciparum* specific factors (within-host competition, natural selection, parasite fitness), human immunity, transmission intensity, female *Anopheles* mosquito factors and transmission blocking mechanisms.

### 3.3. Risk Factors for the Emergence of Antimalarial Resistance Identified by Mathematical Models

Mathematical modeling was identified to explore the dynamics of the emergence of antimalarial resistance, taking into account antimalarial treatment use within the host population in nine articles [8,17,18,19,20,21,22,23,24]. The emergence of resistance was predicted to increase in response to:

- An increase in the proportion of the population who receive antimalarial treatment (population coverage) [17,18];
- A shorter duration of high-dose active pharmaceutical ingredient (API) [22];
- A lower drug efficacy [22];
- Residual drug concentrations (sub-optimal API) [8,23];
- The use of monotherapies compared to combination therapy [18,24];
- Treatment timing [19,22];
- A higher *P. falciparum* mutation rate [22];
- A lower metabolic cost of resistance in *P. falciparum* [22]; and
- A decrease in host natural immunity (corresponding to areas of lower transmission intensity or endemicity) [21,23].

#### 3.3.1. Drug Selection Pressure

Antimalarial use was found to be a major contributor to the emergence of resistance [8,18]. The blood stage replication of *P. falciparum* parasites was identified to be the most likely lifecycle stage for the emergence of resistance, predicted to be up to five times higher in the presence of antimalarials, especially in areas of low transmission [8]. The exposure of *P. falciparum* parasites to residual drug concentrations (sub-optimal API) was also identified to be an important risk factor for selecting resistance [8,23], especially in areas of high transmission [8].

The emergence of resistance was predicted in response to an increase in the population treatment coverage and the use of higher doses (API). This was observed through the modeled use of pyrimethamine (monotherapy), with the model findings recommending that the administration of high doses should be restricted to less than 25% of the population [17].

The use of combination therapy was modeled in three articles and is predicted to decrease the emergence of resistance [18,22,24]. This decrease assumes that (i) both resistances are initially rare; (ii) genetic recombination between resistant genes occurs; and (iii) there is low treatment coverage. However, if no recombination between resistance genes is permitted within the model, combination therapy was not found to be advantageous [18].

The choice of partner drug was identified to be an important factor in prolonging the therapeutic usefulness of combination therapies and preserving the reduction in the chance of the emergence of resistance. Once resistance to one of the partner drugs emerges, there is a decrease in the ability of the combination therapy to delay the emergence of resistance to the other drug. Additionally, the use of a partner drug that had been used as a monotherapy, or one to which the parasite population had been previously exposed, predicted an increase in the time to emergence of resistance [24]. Further, the addition of a partner drug to an already failing treatment did not significantly increase the useful therapeutic life.

Antimalarial dose was found to impact the predicted time to the emergence of resistance. The modeled use of low-dose mefloquine (monotherapy) predicted a faster emergence of resistance than a high dose. The use of low dose resulted in more parasites at all times and increased the opportunity for the selection of mutant *P. falciparum* parasites [23].

#### 3.3.2. *Plasmodium falciparum*

An increase in *P. falciparum* mutation rates was identified to decrease the time to the emergence of resistance; however, the mutation rate is thought to be independent of treatment type or use [5]. An increase in the *PfEMP1* var gene switching was also identified to increase the time to the emergence of resistance [19].

#### 3.3.3. Host Immunity

Antimalarial drugs act synergistically with the host immune defenses [6], and when taking this into account, the timing of treatment was found to be critical for the prevention of the emergence of resistant parasites [19]. A within-host model predicted an increase in the likelihood of a mutant parasite developing into a viable drug-resistant population as a result of early treatment before parasite density triggers anti-PfEMP1 (immune) response, and a high antibody threshold with a long lag time between antibody simulation and activity [19]. In contrast, a stochastic model suggested that early treatment predicted a decrease in the probability of emergence [22].

#### 3.3.4. Transmission Intensity

The acquisition of host immunity occurs through repeated exposure to malaria. In areas of high transmission intensity (endemicity), a greater proportion of the population is thought to have a higher level of immunity [5]. This increase in host immunity results in more asymptomatic infections, which in turn, results in less antimalarial use within the population, decreasing the drug selection pressure [4,52]. The emergence of resistance was predicted to be higher in areas of low transmission than high transmission [8]. In areas with greater levels of host immunity, it was predicted that even when there were low levels of resistance present, the use of low antimalarial dose (API) resulted in more patients being cured [23].

The effect of projected climate change was identified to have an indirect impact on the time to the emergence of resistance, by increasing the transmission intensity, which results in an increase in host immunity (and hence, a decrease in drug selection pressure) [21].

#### 3.3.5. Vector Control

The use of insecticides to control mosquito populations in low transmission areas were predicted to decrease the time to the emergence of resistance, when used in conjunction with antimalarial drugs. However, in high transmission settings, these same interventions were predicted to enhance the emergence of resistance within the population [21].

### 3.4. Risk Factors for the Transmission of Antimalarial Resistance Identified by Mathematical Models

Mathematical modeling was identified to explore the dynamics of the transmission of antimalarial resistance taking into account antimalarial treatment use within the host population in 19 articles [6,25,26,27,28,29,30,31,32,33,34,35,36,37,38,39,40,41,42]. The transmission of resistance was predicted to increase with:

- An increase in the proportion of the population who receive antimalarial treatment (population coverage) [6,27,35,36,38], including intermittent preventive treatment [6] and mass drug administration strategies [35,36];
- The use of monotherapies compared to combination therapy [35,36,37,42];
- An increase in presumptive antimalarial use [37];
- A longer drug half-life [6,41];
- Residual drug concentrations [29,37];
- An increase in the infectious periods of hosts [28];
- A decrease in the recovery rate of nonimmune humans infected by resistant parasites [27];
- Increasing level of resistance (i.e., the number of mutated alleles conferring resistance, partial- or full-resistance) [29,40];
- A decrease of within-host competition between drug-resistant and drug-sensitive *P. falciparum* parasites [32];
- A decrease in the fitness cost associated with resistance [25,27,30,32];
- An increase in the lifespan of resistant-infected mosquitoes [27];
- An increase in mosquito diffusion [27];
- An increase in the number of sporozoites injected from an infected female *Anopheles* mosquito to susceptible human per blood meal [34]; and
- A decrease in the use of transmission blockers (e.g., bednets) [36].

#### 3.4.1. Drug Selection Pressure

An increase in the proportion of antimalarial use was identified as the most important predictor of the transmission of resistance [34]. The existence of a treatment threshold was explored, which was determined by the rates of infection and the infectious periods of resistant and sensitive infections in treated and untreated populations, and findings suggest that resistance will spread and become fixed within the population once the treatment coverage is greater than the threshold [33]. The occurrence of treatment failure (the inability to clear parasites or resolve clinical symptoms of malaria, despite antimalarial treatment administration [4]) was identified as a predictor of the transmission of resistance [37]. Late clinical failure or late parasitological failure as a result of resistance was found to increase the time an individual remained infectious by a few months, whereas low parasite clearance was only found to increase this by a few days [39]. The use of antimalarial treatment was identified to reduce the average duration of sensitive and partially resistant infections [41].

The continued use of monotherapies for which resistance was already established within the population, resulted in an increase in the proportion of resistant infections and the prevalence of malaria infections [36]. The use of ACTs resulted in a long-term reduction in the overall incidence of malaria [35] and the transmission of resistance [37]. The pharmacokinetic properties of partner drugs in ACTs were identified to be the most important determinant of treatment outcome, followed by the rate of parasite growth [42]. For example, an increase in artemisinin IC_50_ (the concentration required to decrease parasitaemia by half) resulted in an increase in resistance, either due to reduced protection to the partner drug or an increase in treatment failure [42].

Partner drug resistance was found to reduce the effectiveness of combination therapies. The use of ACTs (treatment or MDA) in an area with high levels of artemisinin resistance predicted a decrease in treatment effectiveness [35]. Should the use of good quality ACT continue at a high level within the population, even in the presence of artemisinin or partner drug resistance, the model predicted that malaria elimination was possible (note: artemisinin resistance increased to 82–100% of infections prior to malaria elimination) [36].

The use of intermittent prophylaxis administration (IPT) was identified to increase the transmission of resistance, more so in infants than adults (i.e., pregnant women) [6]. Mass drug administrations (MDA) were also identified to increase the transmission of resistance [35] and were not identified to be an effective treatment strategy (alone) to eradicate malaria [36].

The transmission of resistance was predicted to increase with an increase in half-life [6,41]. Similarly, the transmission of resistance was found to increase with mismatched half-lives of partner drugs used in combination therapies [37].

The proportion of human population with residual drug levels was identified to be a driver of the transmission of resistance in high transmission areas, providing the resistant parasites with a survival advantage [37]. In the presence of residual concentrations, highly mutated parasites conferring sulfadoxine-pyrimethamine resistance were predicted to recrudesce earlier. In contrast, when there were fewer mutations, exposure to residual sulfadoxine-pyrimethamine concentrations resulted in sub-patent parasitemia levels (<10 parasites/mL) in the weeks following treatment, which allowed the host’s specific immune response to further control parasitaemia levels, delaying the recrudescence of clinical symptoms [29].

The transmission of resistance was dependent on the transmission rates of sensitive and resistant infections (the ratio of the infectious periods of treated and untreated infections). If the host becomes immediately non-infectious (cannot transmit gametocytes) following treatment, an increase in population treatment coverage results in a decrease in the number of sensitive infections, and an increase in the transmission of resistance. If the host remains infectious post-treatment (still able to transmit gametocytes), the transmission of resistance is dependent on the basic reproduction rate (R_0_) of sensitive and resistant infections [28].

#### 3.4.2. *Plasmodium falciparum*

The speed of transmission is predicted to increase as the basic reproduction rate (R_0_) of drug-resistant parasites becomes larger than the R_0_ of drug-sensitive parasites [27]. The biological cost of resistance (fitness cost) was identified to be less important in predicting the transmission of resistance, than the duration of drug-resistant infections [32]. The forces of intra-host selection and parasite fitness were found to work in opposite directions, and change in magnitude as the frequency of resistance increases. When there were low levels of resistance within the population, the net force increased, resulting in an increase in resistance levels. As resistance frequency increased, the net force decreased and eventually become negative. This net force drives the stabilization of resistance (below 100%) within the population (when the net force equals zero). Under drug selection pressure, the balance of forces was tipped towards the transmission of resistance, which reached 100% [30].

The more tolerant the mutant parasite is to the drug, the less the drug will kill [40], resulting in a greater competitive release compared to the drug-sensitive parasites. The degree of resistance is determined by the concentration of antimalarial drug that the parasite can survive, and may be encoded by one or multiple mutations. Increasing levels of resistance, commonly identified by the number of mutations present that confer resistance, were identified to influence the potential for the transmission of resistance [29]. When resistance was encoded by a mutation at one locus, additional mutated loci were predicted to be a disadvantage [25].

In areas of high treatment coverage (80% of clinical infections treated), the ability for resistant parasites to outcompete and dominate the infection decreases with increasing transmission. In areas of low transmission intensity, resistant parasites were predicted to dominate at all fitness costs. However, as transmission intensity increases, there is a decrease in the range for which the drug-resistant parasites dominate. In areas of low treatment coverage (20% of clinical infections treated), coexistence between resistant and sensitive parasites occurs in areas of low transmission intensity (but resistant parasites do not exclude sensitive), and once again, the range of fitnesses for which coexistence is predicted to occur decreases with increasing transmission intensity [32].

As treatment coverage increased, the proportion of infections with an intermediate level of resistance was predicted to increase [40]. The investigation of a range of dihydrofolate reductase (DHFR) mutations, associated with sulfadoxine-pyrimethamine resistance, predicted the highest transmission potential in moderately-resistant parasites due to a delay in recrudescence and clinical symptoms. However, when exposed to residual concentrations, it was identified that highly-mutated parasites recrudesced earlier [29].

#### 3.4.3. Host Immunity

Host immunity was identified to be a predictor of the transmission of resistance [6,27,28]. Partial immunity was predicted to increase the R_0_ of sensitive parasites [28] and reduce the disease incidence (due to more asymptomatic infections) [28]; however, transmission from asymptomatic infections still continued [28]. An increase in the transmission of resistance was also predicted as a result of the increase in the recovery time of non-immune resistant-infected humans [27].

#### 3.4.4. Transmission Intensity

The degree of transmission intensity (endemicity) in an area was not identified to be predictive of the transmission of resistance [27,28]; however, it is suggested to indirectly influence it through its influence on the acquisition of host immunity and corresponding antimalarial use [26,31]. In areas of low transmission intensity, an increase in drug selection pressure is observed, due to more symptomatic infections requiring treatment (greater population treatment coverage or drug selection pressure). This resulting increase in treatment is suggested to drive the observed difference between low and high transmission intensity areas. As a result of the drug selection pressure observed in areas of low transmission intensity, drug-resistant parasites were predicted to spread and eventually dominate the drug-sensitive parasites [32], and hence, the transmission of resistance was predicted to be greater compared to areas of high transmission intensity [37]. Low transmission intensity areas or areas of unstable transmission were also predicted to coincide with outbreaks of resistance [31].

Other models identified that areas of high transmission intensity were a reservoir for sensitive parasites [31] due to the increase in asymptomatic infections, and corresponding to less drug pressure [26]. This finding held true when the R_0_ of resistant parasites was greater than the R_0_ of sensitive parasites, as the population-wide immunity helped to prevent the transmission of resistance [31]. Another model predicted that partially-resistant parasites were more prevalent in low transmission intensity areas, and fully-resistant parasites in high transmission intensity areas [6].

When areas of intermediate transmission intensity were added to the models, a non-monotonic pattern was observed. In this situation, drug-resistant parasites were favored in areas of low and high transmission intensity, and drug-sensitive parasites in areas of intermediate transmission intensity. The model exhibited a second threshold for the re-establishment of resistance, determined by the cost of resistance, drug coverage, and the fitness of resistant parasites under treatment [26].

Asymptomatic infections, in combination with high transmission intensity, allowed for sulfadoxine-pyrimethamine-resistant parasites (three DHFR mutations and less in DHPS conferring sulfadoxine-pyrimethamine resistance) to expand largely unnoticed [29]. This finding was supported in two other models, which predicted that resistant parasites were more likely to be transmitted in high transmission areas [6,34].

#### 3.4.5. Vector Control

The mosquito factors for which the transmission of resistance was dependent on were: the death rate of mosquitoes infected with resistant parasites [27], the diffusion of mosquitoes coefficient [27], the number of sporozoites per mosquito [34], and the use of transmission blockers, such as insecticide-treated bednets (ITNs) [36]. The use of ITNs, in combination with good quality ACT with high treatment coverage, reduced transmission by 30%, and reduced the time to eradication of malaria by 50% [36].

### 3.5. Risk Factors for the Emergence and Transmission of Antimalarial Resistance Identified by Mathematical Models

Mathematical modeling was identified to explore the dynamics of the emergence and transmission of antimalarial resistance taking into account antimalarial treatment use within the host population in nine articles [43,44,45,46,47,48,49,50,51]. The emergence and transmission of resistance is predicted to increase with:

- An increase in the proportion of the population who receive antimalarial treatment (population coverage) [43,50,51];
- Longer drug half-lives [43];
- Residual drug concentrations [45];
- An increased rate of parasite mutation [43];
- An increased relative fitness of resistant *P. falciparum* parasites compared to their drug-sensitive counterparts [44];
- A decrease in transmission intensity [47]; and
- The decreased use of transmission blockers (i.e., bednets) [49].

#### 3.5.1. Drug Selection Pressure

An increase in the use of antimalarial treatments was predicted to increase the establishment of resistance within the population [50]. The predicted time to the emergence and establishment of resistance was predicted to halve in response to double the amount of antimalarial use [50]. Model findings suggested that the emergence of partial resistance was primarily determined by the proportion of infections treated [45,50] and the proportion with residual drug levels within the population [45]. The emergence of full resistance was primarily determined by the proportion of infections treated [45]. It was shown that resistance to the antimalarial treatment first given will emerge and transmit, and will only decrease if treatment changes [44].

Treatment influences the transmission of resistance, with a trade-off between slowing resistance and curbing disease incidence [47]. The rate of transmission of resistance was predicted to increase in response to an increase in antimalarial use [50]; however, treatment coverage primarily affected disease prevalence [47] and treatment efficacy primarily affected the transmission of resistance [47]. The transmission rate of partially-resistant parasites was predicted to be slower than fully-resistant parasites; however, partially-resistant parasites were more likely to be initially present at higher frequencies [45]. The classical requirement of having R_0_ < 1 to prevent the transmission of malaria was not upheld when multiple levels of resistance were present within the population, suggesting that an increase in treatment has a limited benefit, especially in high transmission settings [51].

The use of combination therapy was predicted to decrease the emergence and transmission of resistance [44,49]. However, the use of a combination therapy to which resistance to one of the partner drugs was already established, resulted in an increased time for the establishment and transmission of resistance within the population [49]. MDA efficacy was predicted to decrease with an increase in the population treatment coverage [49].

#### 3.5.2. *Plasmodium falciparum*

There is evidence of a threshold for determining which strain will dominate, which is dependent on the fitness of the resistant strain, the infection period and treatment rate [44]. The continued treatment with an ineffective antimalarial to which resistance has emerged is predicted to increase the proportion of infections in each class of resistance present [51].

#### 3.5.3. Transmission Intensity

The proportion of resistant infections was predicted to be lower in areas of high transmission intensity than low transmission intensity [47].

#### 3.5.4. Vector Control

The modeled use of transmission blockers provided mixed results. A decrease in the emergence and transmission of resistance was predicted in response to an increase in ITN use in one model [49]; however, the use of transmission blockers (e.g., bednets and vaccination programs) was not predictive of the emergence of resistance in another [45]. It was further suggested that the use of transmission blockers may be indirectly related to the emergence of resistance through the corresponding reduction of antimalarial use associated with transmission blockers [45].

## 4. Discussion

The scoping review identified 37 articles containing mathematical models exploring the dynamics of the emergence and/or transmission of antimalarial resistance, taking into account the use of antimalarial treatment in the host (human) population. The model findings identified that the drug selection pressure exerted by the use of antimalarial treatments plays an important role in the emergence and transmission of resistance. Further, commonly-identified drivers were the proportion of antimalarial use within the population and residual drug levels (sub-optimal/sub-therapeutic API). Factors that contribute to a greater drug selection pressure on the system are of importance for the assessment of their impact, and for determination of areas of intervention.

### 4.1. Mathematical Models

A range of mathematical models was used to explore the dynamics of the emergence and/or transmission of antimalarial resistance. Deterministic modeling was the most common type (68%), with an additional three articles (8%) using a combination of deterministic and stochastic modeling techniques.

An analysis of model behavior, sensitivity of parameters, and validation of model outputs were presented in over half of the manuscripts, which are vital for understanding the stability of the model and reliability of the findings. The model assumptions were provided in all the articles reviewed. The transparency and reproducibility of the mathematical models allow for rigorous review of the modeling methods utilized, underlying assumptions and a more in-depth understanding of the inputs that drive the model dynamics and their limitations. We observed a general increase in the transparency and reproducibility of models over time, with the inclusion of model equations, parameter values, and model flow diagrams within the manuscripts (or Supplementary Materials).

There is a tradeoff between the complexity of a mathematical model and the interpretation of results. Model complexity was added through the populations modeled and the factors explored. The nature of the emergence of resistance as a mutation event within the *P. falciparum* parasite followed by natural selection and drug selection within the human host, suggests the need for modeling the interactions between the human and *Plasmodium* parasite population, as was the case in 56% (5/9) of the emergence articles reviewed. The transmission of malaria occurs between the human and mosquito populations, through *P. falciparum* gametocytes (human-to-mosquito transmission) and *P. falciparum* sporozoites (mosquito-to-human transmission). The human population only was modeled in 42% (8/19) of the transmission models, where the transmission to and from the mosquito was summarized within the equations. However, the human and mosquito populations were modeled in 32% (6/19) of the transmission models reviewed. When modeling the impact of antimalarial use on the emergence and transmission of resistance, the human and mosquito populations were modeled in 44% (4/9) articles reviewed, and the human population only in 33% (3/9). The added complexity of modeling all three populations (human, mosquito and *P. falciparum*) was not common at 14% (5/37) across the three model categories.

As we have observed in this review, there are many factors that impact the emergence and transmission of antimalarial resistance in the human population, many of which are impacted by, or predict the effect of, drug use in the population (e.g., natural immunity, transmission intensity).

### 4.2. Risk Factors for the Emergence and Transmission of Resistance

An increase in the proportion of the population that receives antimalarial treatment (population treatment coverage) predicted an increase in the emergence and/or transmission of resistance. Risk factors contributing to the proportion of the population receiving antimalarial treatment are:

- Antimalarial treatment to resolve clinical symptoms [6,17,18,27,35,36,38,43,50,51];
- Use of intermittent preventive treatment (IPT) [6];
- Presumptive treatment [37]; and
- MDAs and mass screening and treatments (MSATs) [35,36].

The exposure of parasites to residual drug concentrations (sub-optimal or sub-therapeutic API) was identified as a driver of the emergence of antimalarial resistance, and to increase the transmission of resistance. Risk factors contributing to the exposure of parasites to residual drug concentrations are:

- The presence of residual drug concentration [8,23,29,37,45];
- Long half-lives [6,41,43];
- Low antimalarial dose (API) [23];
- Low drug efficacy [22]; and
- Shorter duration of high dose (API) [22].

Other treatment factors include the timing of antimalarial treatment [19,22] and the use of combination therapies. The use of combination therapies was consistently predictive of a decrease in the emergence and transmission of resistance [18,24,35,36,37,42], provided the treatment had not previously been used as a monotherapy [24] and resistance had not emerged to one of the partner drugs [35,49]. This finding highlights the importance of the adherence to Antimalarial Treatment Guidelines proposed by the World Health Organization [53].

Other risk factors for the emergence and transmission of resistance that were included in the models reviewed can be categorized into the following groups:

- *P. falciparum* population: mutation rate [22,43], fitness cost of resistance [22,25,27,30,32,44], degree of resistance [29,40], within-host competition [32];
- Human population: infectious period [28]; recovery rate of non-immune humans infected with resistant parasites [27]; and natural immunity [21,23] (related to transmission intensity [47]); and
- Female *Anopheles* mosquito population: lifespan of resistant-infected mosquitoes [27]; mosquito diffusion [27]; number of sporozoites [34]; and transmission blockers (e.g., bednets) [36,49].

### 4.3. Review Limitations

The primary limitation in the search strategy was the restriction of articles to English-language only studies. This excludes any relevant literature published in other languages. The majority of the studies reviewed were theoretical (76%), and as such, highlighted the limitations often experienced with a lack of comprehensive datasets in this field [33].

### 4.4.Implications for Further Research

The scoping review has highlighted areas requiring further research and model development, in order to better understand the impact of antimalarial use on the emergence and transmission of resistance.

#### 4.4.1. Drug Selection Pressure

Antimalarial treatments that contribute to the overall population treatment coverage are of importance. The use of IPT, MDA and MSAT strategies are areas of research that need to be evaluated further mathematically, in order to understand the impact of population treatment coverage on the emergence and/or transmission of resistance.

Antimalarial treatment that contributes to the presence of residual drug concentrations within the population is of importance. The quality, efficacy and percentage API (including high/low dose, residual API, self-medication, non-compliance) are key areas of research that need to be evaluated further mathematically, in order to understand the impact of residual concentrations on the emergence and/or transmission of resistance.

Although the effect of poor drug quality (substandard, falsified and/or degraded medicines) was not addressed in the mathematical models reviewed, it is being increasingly recognized as a source of residual drug concentrations [54]. Approximately 30% of antimalarial medicines in Africa and Asia are considered to be substandard or falsified [55,56]. Substandard antimalarials are genuine medicines that generally meet the API and/or formulation content guidelines but fail to meet good manufacturing practice [54]. Degraded antimalarials leave the manufacturer as quality-assured medicines but degrade due to inadequate storage conditions (excess heat, humidity or exposure to light) [53,57]. Both substandard and degraded antimalarials can be a source of sub-therapeutic API concentrations. Falsified medicines are fraudulently mislabeled with respect to identity and/or source [54,58,59], the quality and manufacture of which are not regulated by government health sectors to ensure internationally recognized standards are met. The API may be correct or incorrect with regards to ingredient and dose, or they may contain incorrect ingredients [60]. As a result of poor formulation techniques, falsified medicines may release an incorrect amount of API and expose parasites to sub-therapeutic API concentrations. The impact of the use of poor-quality antimalarial medicines on the emergence and transmission of resistance needs to be explored as they may be a significant contributor to the drivers of resistance identified in this review: population treatment coverage and residual concentrations.

The practice of self-diagnosis (self-medication) was also not addressed in the mathematical models reviewed. This practice can result in the patient having the wrong treatment regimen, a shorter treatment period than is recommended, and/or an incorrect dose of API(s) (poor quality antimalarials, or quality-assured antimalarials that have expired or degraded). Self-medication is commonly identified to be a contributor to the population treatment coverage, without a malaria diagnosis [4]. As the API declines with time, this provides a selective pressure on any future infections, adding to the residual concentrations within the population. These areas of future research are outlined in Table 2.

The lifecycle of the *P. falciparum* parasite has stages within the human host and the female *Anopheles* vector. In order to fully understand the impact of antimalarial use on the emergence and transmission of *P. falciparum* drug resistance in humans, we propose that a review of the effect of drug use on *Plasmodium* spp. parasite populations needs to be carried out. The combination of this scoping review with the proposed review would provide a deeper understanding of the underlying dynamics.

#### 4.4.2. Influencing Factors

The infectivity of gametocytes following treatment, taking into account the antimalarial treatment and quality, must be explored in order to fully understand how the transmission of resistance is affected by antimalarial use.

Conflicting findings of the impact of natural immunity, transmission intensity, and transmission blockers on the emergence and transmission of resistance were revealed in this review, and provide areas for further research.

## 5. Conclusions

The emergence and transmission of resistance to antimalarial treatments are continuing to hamper malaria elimination efforts. This article has reviewed the use of mathematical models to describe the relationship between antimalarial use and *P. falciparum* antimalarial resistance within the human population, and identified other factors that influence this relationship. However, this scoping review has identified key areas for future research in order to better understand the impact of antimalarial use on the emergence and transmission of antimalarial resistance.

## Figures and Tables

**Table 1:** A summary of the model features.

Model Feature	Frequency [Reference(s)]		
	Emergence (Nine Articles)	Transmission (19 Articles)	Both (Nine Articles)
**Model Description**			
Model type:			
Deterministic	6 [8,18,19,20,23,24]	13 [6,25,26,27,28,31,33,35,38,39,40,41,42]	6 [43,44,45,48,49,51]
Stochastic	3 [17,21,22]	5 [29,30,32,34,37]	1 [46]
Both		1 [36]	2 [47,50]
Scope of model:			
Applied	2 [17,23]	4 [35,36,39,42]	3 [43,49,51]
Theoretical	7 [8,18,19,20,21,22,24]	15 [6,25,26,27,28,29,30,31,32,33,34,37,38,40,41]	6 [44,45,46,47,48,50]
Populations modeled:			
Human		8 [6,30,31,35,36,37,39,41]	3 [48,49,50]
Human & mosquito	2 [17,18]	6 [26,27,28,33,34,40]	4 [44,46,47,51]
Human & plasmodia	5 [8,19,20,23,24]	3 [25,29,42]	1 [45]
Human, mosquito & plasmodia	2 [21,22]	2 [32,38]	1 [43]
Transparency and reproducibility of model:			
Assumptions	9 [8,17,18,19,20,21,22,23,24]	19 [6,25,26,27,28,29,30,31,32,33,34,35,36,37,38,39,40,41,42]	8 [43,44,45,46,47,48,49,50,51]
Equations provided	7 [8,19,20,21,22,23,24]	17 [6,26,27,28,30,31,32,33,34,35,36,37,38,39,40,41,42]	8 [43,44,45,46,47,48,50,51]
Model flowchart provided	6 [8,17,18,19,21,22]	18 [6,26,27,28,29,31,32,33,34,35,36,37,38,39,40,41]	5 [43,46,48,49,51]
Model validation	2 [17,23]	5 [26,27,29,35,42]	2 [43,45]
Parameters provided	8 [8,17,19,20,21,22,23,24]	16 [6,26,27,28,30,31,32,34,35,36,37,38,39,40,41,42]	8 [43,44,45,46,47,49,50,51]
Sensitivity analysis	2 [19,21]	5 [26,29,30,36,37]	2 [47,50]
**Antimalarial Resistance**			
Monotherapy			
Artemisinin	1 [8]	3 [35,39,42]	1 [49]
Artesunate		3 [6,35,36]	1 [43]
Atovaquone			1 [49]
Chloroproguanil-dapsone		1 [41]	
Chloroquine		1 [42]	1 [43]
Lumefantrine		1 [42]	
Mefloquine	1 [23]	1 [42]	
Piperaquine		5 [6,35,36,39,42]	
Pyrimethamine	1 [17]		
Quinine			1 [43]
Not specified	2 [18,22]	3 [25,27,34]	
Combination therapy			
Artemisinin-based combination therapy (ACT)		1 [39]	1 [50]
Sulfadoxine-pyrimethamine	1 [24]	4 [25,29,39,41]	2 [43,45]
Not specified	2 [18,22]	2 [25,34]	
Partner-drug resistance (not specified)	2 [8,24]	3 [34,35,37]	
Resistance type not specified	2 [19,20]	8 [26,28,30,31,32,33,38,40]	5 [44,46,47,48,51]
Degree of resistance specified (partial, full)	3 [21,23,24]	5 [6,29,39,40,41]	3 [45,46,51]
**Drug Selection Pressure**			
Antimalarial treatment:			
Monotherapies			
Artemisinin	1 [8]		
Artesunate		1 [36]	2 [43,49]
Chloroproguanil-dapsone	1 [24]	2 [6,41]	
Chloroquine		1 [42]	1 [43]
Lumefantrine		1 [42]	
Mefloquine	1 [23]	1 [42]	
Piperaquine		1 [42]	
Pyrimethamine	1 [17]		
Quinine			1 [43]
Not specified	2 [18,22]	3 [25,27,34]	
Combination therapies			
Artemether-lumefantrine		1 [42]	
Artemisinin-based combination therapy (ACT)	1 [8]	1 [37]	2 [49,50]
Artemisinin-piperaquine		1 [35]	
Artemisinin-piperaquine + primaquine		2 [35,36]	1 [49]
Artesunate + piperaquine		1 [36]	
Artesunate + mefloquine		1 [42]	
Artesunate + chloroquine		1 [42]	
Artesunate-lumefantrine		1 [42]	
Atovaquone + progunail		1 [36]	1 [49]
Atovaquone + progunail + primaquine		1 [36]	1 [49]
Chloroproguanil-dapsone + artesunate	1 [24]		
Dihydroartemisinin + piperaquine		2 [39,42]	
Sulfadoxine-pyrimethamine	1 [24]	3 [6,29,41]	2 [43,45]
Sulfadoxine-pyrimethamine + amodiaquine	1 [24]		
Sulfadoxine-pyrimethamine + artesunate	1 [24]	1 [39]	1 [45]
Chloroproguanil-dapsone + artesunate			1 [45]
Not specified	2 [18,22]	2 [25,34]	
Treatment not specified	3 [19,20,21]	8 [26,28,30,31,32,33,38,40]	5 [44,46,47,48,51]
Antimalarial treatment strategies:			
Intermittent-preventive treatment (IPT)		3 [6,39,41]	1 [49]
Mass drug administration (MDA)		2 [35,36]	1 [49]
Mass screening and treatment (MSAT)		1 [36]	
Antimalarial properties and duration of treatment:			
Full/partial treatment duration		1 [25]	
Half-life/decay of concentration with time	2 [22,23]	3 [6,41,42]	2 [43,45]
High/low dose	2 [22,23]		1 [49]
Residual levels	2 [8,23]	1 [41]	
Levels of drug efficacy	1 [17]		
Parasite growth restriction following treatment	1 [22]		
Patient compliance		1 [37]	
Protection from reinfection	1 [24]		
Transmissibility following treatment	1 [22]		
**Potential Influencing Factors**			
*Plasmodium falciparum*:			
Asexual parasite density	3 [17,22,23]	1 [29]	
Epistasis		1 [25]	
Frequency of mutation		1 [25]	
Gametocyte parasite density	3 [17,22,24]	1 [29]	
Genetic recombination	3 [18,20,22]	2 [25,34]	
Inbreeding and/or random mating		3 [25,34,38]	
Infectivity/transmissibility following treatment	1 [17]	4 [28,29,31,34]	
Parasite fitness	4 [19,20,21,22]	12 [6,25,26,27,30,31,32,33,34,38,40,41]	6 [44,46,47,48,50,51]
Multiplicity of infection (MOI)		1 [32]	
Mutation rate	1 [22]		
Natural selection	1 [22]	3 [26,30,32]	1 [46]
Host immunity:			
Acquired/clinical immunity or host age-dependent	4 [8,17,21,23]	13 [6,25,26,27,28,31,33,35,36,39,40,41,42]	3 [43,46,49]
Immune response	1 [19]		1 [47]
Generalized/strain specific immunity	1 [20]		1 [47]
Symptomatic and/or asymptomatic infection	1 [22]	7 [6,29,31,35,36,39,41]	
Transmission intensity	3 [8,21,24]	11 [6,25,26,31,32,36,37,38,39,41]	1 [45]
Female *Anopheles* mosquito:			
Competition for blood meal			1 [43]
Entomological inoculation rate (EIR)		5 [31,32,37,41,42]	
Fitness of mosquitoes to produce offspring	1 [21]		
Insecticide resistance	1 [21]		
Population size dependent on climatic factors	2 [17,21]	1 [26]	
Sporozoite measure (count/rate)		1 [34]	
Transmission blockers:			1 [45]
Insecticidal bednets		3 [35,36,39]	1 [49]
Transmission potential	1 [21]	1 [26]	
Vectorial capacity		4 [26,31,32,37]	

**Table 2:** Areas of future mathematical modeling research that contribute to the main drivers of the emergence and transmission of resistance identified in the scoping review, where gaps are indicated by ‘X’.

Treatment Scenario	Emergence	Transmission	Emergence and Transmission
Contributing to population treatment coverage			
IPT use	X		
MDA	X		
MSAT	X		X
Self-medication	X	X	X
Contributing to residual drug concentrations			
Drug efficacy		X	X
Drug quality (falsified, substandard and degraded)	X	X	X
Full/partial treatment and patient compliance	X		X
High/low dose		X	
Percentage API	X	X	X
Residual/subtherapeutic API			X
Self-medication	X	X	X

## Supplementary Materials

The following are available online at http://www.mdpi.com/2414-6366/2/4/54/s1, Table S1: Medline and Embase Database Search, Table S2: CINAHL Database Search, Table S3: Web of Science Database Search, Table S4: Cochrane Library Database Search, Table S5: Scopus Database Search, and Table S6: Grey Literature Search (Google Scholar and Open Grey).
