# Supplementary material for: The Impact of Antimalarial Use on the Emergence and Transmission of Plasmodium falciparum Resistance: A Scoping Review of Mathematical Models
Source: Trop Med Infect Dis. 2017 Oct 15;2(4):54. doi: 10.3390/tropicalmed2040054 (PMC6082068; doi:10.3390/tropicalmed2040054)
Supplement: Supplementary file 1 [file tropicalmed-02-00054-s001.pdf]

Supplementary Materials: The Impact of Antimalarial Use on the Emergence and Transmission of *Plasmodium falciparum* Resistance: A Scoping Review of Mathematical Models

Aleisha R. Brock, Carole A. Gibbs, Joshua V. Ross and Adrian Esterman

S1. Medline and Embase Database Search

Table S1. Search strategy for Medline and Embase.

Search Line	Search Term
1	malaria/
2	malaria falciparum/
3	Plasmodium falciparum/
4	Plasmodium/
5	gametogenesis/
6	(malaria or plasmodium or plasmodia or marsh fever or fever, marsh or paludism or falciparum or gametogeneses or gametogenesis or gametocyte or gametocytocidal).ti,ab.
7	or/1-6
8	drug resistance/
9	treatment failure/
10	drug tolerance/
11	treatment outcome/
12	(failure*, treatment or treatment failure* or drug resistance or resistance, drug or clinical effectiveness* or clinical efficacy or effectiveness*, clinical or effectiveness, treatment or efficacy, clinical or efficacy, treatment or outcome, patient-relevant or outcome, rehabilitation or outcome*, treatment or patient relevant outcome or patient-relevant outcome* or rehabilitation outcome or treatment effectiveness or treatment efficacy or treatment outcome or drug tolerance* or tolerance*, drug).ti,ab.
13	or/8-12
14	exp Antimalarials/
15	drug therapy/
16	drug therapy, combination/
17	counterfeit drugs/
18	self medication/
19	prescription drug misuse/
20	(antimalarial* or Acedapsone or Amodiaquine or Artemisinins or Atovaquone or Chloroquine or Dapsone or Doxycycline or Hydroxychloroquine or Mefloquine or Primaquine or Proguanil or Pyrimethamine or Quinacrine or Quinidine or Quinine or Sulfadoxine or Sulfalene or Trimethoprim or Trimethoprim Sulfamethoxazole Drug Combination or drug therap* or therap*,

	drug or self medicat* or prescription drug misuse* or combination drug therap* or drug polytherap* or drug therap*, combination or polytherap*, drug or therap*, combination drug or counterfeit drug* or counterfeit medicine* or counterfeiting, drug or drug counterfeiting or drugs, counterfeit or drug*, fake or fake drug* or medicine*, counterfeit or fake* or counterfeit* or falsif* or forge* or illicit or quality or medication*, self or self medication* or prescription drug misuse*).ti,ab.
21	((fake* or counterfeit* or falsif* or quality or forge* or illicit) and (drug* or antimalarial*)).ti,ab.
22	or/14-21
23	(ross-macdonald or quantif* or mathemat* or model* study or differential equation*).ti,ab.
24	(stud* adj1 (math* or experiment* or statistic* or theoretic* or binomial or polynomial or probabilistic or genetic* or epidemiolog* or deterministic* or simulat*)).ti,ab.
25	(model* adj1 (math* or experiment* or statistic* or theoretic* or binomial or polynomial or probabilistic or genetic* or epidemiolog* or deterministic* or simulat*)).ti,ab.
26	exp models, theoretical/
27	exp models, statistical/
28	or/23-27
29	and/7,13,22,28

S2. CINAHL Database Search

Table S2. Search strategy for CINAHL.

Search Line	Search Term
S1	MW malaria
S2	TI (malaria or plasmodium or plasmodia or marsh fever or fever, marsh or paludism or falciparum or gametogeneses or gametogenesis or gametocyte or gametocytocidal) OR AB (malaria or plasmodium or plasmodia or marsh fever or fever, marsh or paludism or falciparum or gametogeneses or gametogenesis or gametocyte or gametocytocidal)
S3	S1 OR S2
S4	MW drug resistance
S5	MW treatment Failure
S6	MW drug tolerance
S7	TI (failure*, treatment or treatment failure* or drug resistance or resistance, drug or clinical effectiveness* or clinical efficacy or effectiveness*, clinical or effectiveness, treatment or efficacy, clinical or efficacy, treatment or outcome, patient-relevant or outcome, rehabilitation or outcome*, treatment or patient relevant outcome or patient-relevant outcome* or rehabilitation outcome or treatment effectiveness or treatment efficacy or treatment outcome or drug tolerance* or tolerance*, drug) OR AB (failure*, treatment or treatment failure* or drug resistance or resistance, drug or clinical effectiveness* or clinical efficacy or effectiveness*, clinical or effectiveness, treatment or efficacy, clinical or

	efficacy, treatment or outcome, patient-relevant or outcome, rehabilitation or outcome*, treatment or patient relevant outcome or patient-relevant outcome* or rehabilitation outcome or treatment effectiveness or treatment efficacy or treatment outcome or drug tolerance* or tolerance*, drug)
S8	S4 OR S5 OR S6 OR S7
S9	MW Antimalarials
S10	MW drug therapy
S11	MW drug therapy, combination
S12	MW counterfeit drugs
S13	MW self medication
S14	TI (antimalarial* or Acedapsone or Amodiaquine or Artemisinins or Atovaquone or Chloroquine or Dapsone or Doxycycline or Hydroxychloroquine or Mefloquine or Primaquine or Proguanil or Pyrimethamine or Quinacrine or Quinidine or Quinine or Sulfadoxine or Sulfalene or Trimethoprim or Trimethoprim Sulfamethoxazole Drug Combination or drug therap* or therap*, drug or self medicat* or prescription drug misuse* or combination drug therap* or drug polytherap* or drug therap*, combination or polytherap*, drug or therap*, combination drug or counterfeit drug* or counterfeit medicine* or counterfeiting, drug or drug counterfeiting or drugs, counterfeit or drug*, fake or fake drug* or medicine*, counterfeit or fake* or counterfeit* or falsif* or forge* or illicit or quality or medication*, self or self medication* or prescription drug misuse*) OR AB (antimalarial* or Acedapsone or Amodiaquine or Artemisinins or Atovaquone or Chloroquine or Dapsone or Doxycycline or Hydroxychloroquine or Mefloquine or Primaquine or Proguanil or Pyrimethamine or Quinacrine or Quinidine or Quinine or Sulfadoxine or Sulfalene or Trimethoprim or Trimethoprim Sulfamethoxazole Drug Combination or drug therap* or therap*, drug or self medicat* or prescription drug misuse* or combination drug therap* or drug polytherap* or drug therap*, combination or polytherap*, drug or therap*, combination drug or counterfeit drug* or counterfeit medicine* or counterfeiting, drug or drug counterfeiting or drugs, counterfeit or drug*, fake or fake drug* or medicine*, counterfeit or fake* or counterfeit* or falsif* or forge* or illicit or quality or medication*, self or self medication* or prescription drug misuse*)
S15	TI (fake* or counterfeit* or falsif* or quality or forge* or illicit) AND TI (drug* or antimalarial*)
S16	AB (fake* or counterfeit* or falsif* or quality or forge* or illicit) AND AB (drug* or antimalarial*)
S17	S9 OR S10 OR S11 OR S12 OR S13 OR S14 OR S15 OR S16
S18	TI (ross-macdonald or quantif* or mathemat* or model* study or differential equation*) OR AB (ross-macdonald or quantif* or mathemat* or model* study or differential equation*)
S19	TI stud* AND TI (math* or experiment* or statistic* or theoretic* or binomial or polynomial or probabilistic or genetic* or epidemiolog* or deterministic* or simulat*)
S20	AB stud* AND AB (math* or experiment* or statistic* or theoretic* or binomial or polynomial or probabilistic or genetic* or epidemiolog* or deterministic* or simulat*)

S21	TI model* AND TI (math* or experiment* or statistic* or theoretic* or binomial or polynomial or probabilistic or genetic* or epidemiolog* or deterministic* or simulat*)
S22	AB model* AND AB (math* or experiment* or statistic* or theoretic* or binomial or polynomial or probabilistic or genetic* or epidemiolog* or deterministic* or simulat*)
S23	MW models, theoretical
S24	S18 OR S19 OR S20 OR S21 OR S22 OR S23
S25	S3 AND S8 AND S17 AND S24

S3. Web of Science Database Search

Table S3. Search strategy for Web of Science.

Search Line	Search Term
1	TS=(malaria OR "malaria falciparum" OR "Plasmodium falciparum" OR gametogenesis*)<br>DocType=All document types; Language=All languages;
2	TS=("drug resistance" OR "drug tolerance" OR "treatment outcome" OR "treatment failure")<br>DocType=All document types; Language=All languages;
3	TS=(antimalarial* OR "drug therapy" OR “drug quality” OR ((fake* OR counterfeit* OR falsif* OR quality OR forge* OR illicit) AND (drug* OR antimalarial*)) OR “self-medication”)<br>DocType=All document types; Language=All languages;
4	TS=(“mathematic* model*” OR model OR “theoretic* model*”)<br>DocType=All document types; Language=All languages;
5	#4 AND #3 AND #2 AND #1<br>DocType=All document types; Language=All languages;

S4. Cochrane Library Database Search

Table S4. Search strategy for Cochrane Library.

Search Line	Search Term
1	malaria or "malaria falciparum" or "Plasmodium falciparum" or gametogenesis*
2	"drug resistance" or "drug tolerance" or "treatment outcome" or "treatment failure"
3	antimalarial* or "drug therapy" or "drug quality" or ((fake* or counterfeit* or falsif* or quality or forge* or illicit) and (drug* or antimalarial*)) or "self-medication"
4	"mathematic* model*" or model or "theoretic* model*"
5	#1 and #2 and #3 and #4

S5. Scopus Database Search

Table S5. Search strategy for Scopus.

Search Line	Search Term
1	TITLE-ABS-KEY (malaria OR plasmodium OR plasmodia OR marsh AND fever OR fever, AND marsh OR paludism OR falciparum OR gametogeneses OR gametogenesis OR gametocyte OR gametocytocidal)
2	(TITLE-ABS-KEY ("failure*, treatment" OR "treatment failure*" OR "drug resistance" OR "resistance, drug" OR "clinical effectiveness*" OR "clinical efficacy" OR "effectiveness*, clinical" OR "effectiveness, treatment") OR TITLE-ABS-KEY ("efficacy, clinical" OR "efficacy, treatment" OR "outcome, patient-relevant" OR "outcome, rehabilitation" OR "outcome*, treatment" OR "patient relevant outcome" OR "patient-relevant outcome*") OR TITLE-ABS-KEY ("rehabilitation outcome" OR "treatment effectiveness" OR "treatment efficacy" OR "treatment outcome" OR "drug tolerance*" OR "tolerance*,drug"))
3	(TITLE-ABS-KEY (antimalarial* OR acedapsone OR amodiaquine OR artemisinins OR atovaquone OR chloroquine OR dapsone OR doxycycline OR hydroxychloroquine OR mefloquine OR primaquine OR proguanil OR pyrimethamine OR quinacrine OR quinidine OR quinine OR sulfadoxine) OR TITLE-ABS-KEY (sulfalene OR trimethoprim OR "Trimethoprim Sulfamethoxazole Drug Combination" OR "drug therap*" OR "therap*, drug" OR "self medicat*" OR "prescription drug misuse*" OR "combination drug therap*") OR TITLE-ABS-KEY ("drug polytherap*" OR "drug therap*, combination" OR "polytherap*, drug" OR "therap*, combination drug" OR "counterfeit drug*" OR "counterfeit medicine*" OR "counterfeiting, drug" OR "drug counterfeiting") OR TITLE-ABS-KEY ("drugs, counterfeit" OR "drug*, fake" OR "fake drug*" OR "medicine*, counterfeit" OR fake* OR counterfeit* OR falsif* OR forge* OR illicit OR quality OR "medication*, self" OR self AND medication* OR "prescription drug misuse*") OR TITLE-ABS-KEY (((fake* OR counterfeit* OR falsif* OR quality OR forge* OR illicit) AND (drug* OR antimalarial*))))
4	(TITLE-ABS-KEY (ross-macdonald OR quantif* OR mathemat* OR "model* study" OR "differential equation*") OR TITLE-ABS-KEY ((stud* W/1 (math* OR experiment* OR statistic* OR theoretic* OR binomial OR polynomial OR probabilistic OR genetic* OR epidemiolog* OR deterministic* OR simulat*))) OR TITLE-ABS-KEY ((model* W/1 (math* OR experiment* OR statistic* OR theoretic* OR binomial OR polynomial OR probabilistic OR genetic* OR epidemiolog* OR deterministic* OR simulat*))))
5	(TITLE-ABS-KEY (malaria OR plasmodium OR plasmodia OR marsh AND fever OR fever, AND marsh OR paludism OR falciparum OR gametogeneses OR gametogenesis OR gametocyte OR gametocytocidal)) AND ((TITLE-ABS-KEY ("failure*, treatment" OR "treatment failure*" OR "drug resistance" OR "resistance, drug" OR "clinical effectiveness*" OR "clinical efficacy" OR "effectiveness*, clinical" OR "effectiveness,

treatment") OR TITLE-ABS-KEY ("efficacy, clinical" OR "efficacy, treatment" OR "outcome, patient-relevant" OR "outcome, rehabilitation" OR "outcome*, treatment" OR "patient relevant outcome" OR "patient-relevant outcome*") OR TITLE-ABS-KEY ("rehabilitation outcome" OR "treatment effectiveness" OR "treatment efficacy" OR "treatment outcome" OR "drug tolerance*" OR "tolerance*,drug"))) AND ((TITLE-ABS-KEY (antimalarial* OR acedapsone OR amodiaquine OR artemisinins OR atovaquone OR chloroquine OR dapsone OR doxycycline OR hydroxychloroquine OR mefloquine OR primaquine OR proguanil OR pyrimethamine OR quinacrine OR quinidine OR quinine OR sulfadoxine) OR TITLE-ABS-KEY (sulfalene OR trimethoprim OR "Trimethoprim Sulfamethoxazole Drug Combination" OR "drug therap*" OR "therap*, drug" OR "self medicat*" OR "prescription drug misuse*" OR "combination drug therap*") OR TITLE-ABS-KEY ("drug polytherap*" OR "drug therap*, combination" OR "polytherap*, drug" OR "therap*, combination drug" OR "counterfeit drug*" OR "counterfeit medicine*" OR "counterfeiting, drug" OR "drug counterfeiting") OR TITLE-ABS-KEY ("drugs, counterfeit" OR "drug*, fake" OR "fake drug*" OR "medicine*, counterfeit" OR fake* OR counterfeit* OR falsif* OR forge* OR illicit OR quality OR "medication*, self" OR self AND medication* OR "prescription drug misuse*") OR TITLE-ABS-KEY (((fake* OR counterfeit* OR falsif* OR quality OR forge* OR illicit) AND (drug* OR antimalarial*))))) AND ((TITLE-ABS-KEY (ross-macdonald OR quantif* OR mathemat* OR "model* study" OR "differential equation*") OR TITLE-ABS-KEY ((stud* W/1 (math* OR experiment* OR statistic* OR theoretic* OR binomial OR polynomial OR probabilistic OR genetic* OR epidemiolog* OR deterministic* OR simulat*))) OR TITLE-ABS-KEY ((model* W/1 (math* OR experiment* OR statistic* OR theoretic* OR binomial OR polynomial OR probabilistic OR genetic* OR epidemiolog* OR deterministic* OR simulat*)))))

S6. Grey Literature Search (Google Scholar and Open Grey)

Search Terms: (malaria or plasmodium or plasmodia or marsh fever or fever, marsh or paludism or falciparum or gametogeneses or gametogenesis or gametocyte or gametocytocidal) AND (failure*, treatment or treatment failure* or drug resistance or resistance, drug or clinical effectiveness* or clinical efficacy or effectiveness*, clinical or effectiveness, treatment or efficacy, clinical or efficacy, treatment or outcome, patient-relevant or outcome, rehabilitation or outcome*, treatment or patient relevant outcome or patient-relevant outcome* or rehabilitation outcome or treatment effectiveness or treatment efficacy or treatment outcome or drug tolerance* or tolerance*, drug) AND (antimalarial* or Acedapsone or Amodiaquine or Artemisinins or Atovaquone or Chloroquine or Dapsone or Doxycycline or Hydroxychloroquine or Mefloquine or Primaquine or Proguanil or Pyrimethamine or Quinacrine or Quinidine or Quinine or Sulfadoxine or Sulfalene or Trimethoprim or Trimethoprim Sulfamethoxazole Drug Combination or drug therap* or therap*, drug or self medicat* or prescription drug misuse* or combination drug therap* or drug polytherap* or drug therap*, combination or polytherap*, drug or therap*, combination drug or counterfeit drug* or counterfeit medicine* or counterfeiting, drug or drug counterfeiting or drugs, counterfeit or drug*, fake or fake drug* or medicine*, counterfeit or fake* or counterfeit* or falsif* or forge* or illicit or quality or medication*, self or self medication* or prescription drug misuse* OR (fake* or counterfeit* or falsif* or quality or forge* or illicit) and (drug* or antimalarial*)) AND (ross-macdonald or quantif* or mathemat* or model* study or differential equation* OR stud* near (math* or experiment* or statistic* or theoretic* or binomial or polynomial or probabilistic or genetic* or epidemiolog* or

deterministic* or simulat*)) OR (model* near (math* or experiment* or statistic* or theoretic* or binomial or polynomial or probabilistic or genetic* or epidemiolog* or deterministic* or simulat*))

© 2017 by the authors. Submitted for possible open access publication under the terms and conditions of the Creative Commons Attribution (CC BY) license (http://creativecommons.org/licenses/by/4.0/).
